# Comparative study on consumers’ choice behaviors in selecting pork in rational and irrational scenarios

**DOI:** 10.3389/fpsyg.2022.1016933

**Published:** 2022-10-11

**Authors:** Lingling Xu, Meidan Yu, Xiujuan Chen

**Affiliations:** ^1^Institute for Food Safety Risk Management, School of Business, Jiangnan University, Wuxi, China; ^2^School of Business, Jiangnan University, Wuxi, China

**Keywords:** pork hindquarter, food safety, animal welfare, attribute preference, decoy effect

## Abstract

To better understand the purchasing decision-making process of humane pork, and examine the internal relationship between consumers’ preferences in rational consumption and irrational decoy scenarios, 405 consumers in Wuxi City, Jiangsu Province, and China were surveyed. Attributes were set for breeding time, breeding mode, diet cleanliness label, and price, and the first three among them reflect animal welfare conditions. The results show that in the rational consumption scenarios, consumers pay the most attention to the price attribute, followed by the attribute of diet cleanliness label, breeding mode, and breeding time. In the irrational decoy scenarios, consumers are most likely to be affected by the attribute decoy of diet cleanliness label, which have the utility of avoiding food safety risks. In addition, the decoy effect triggered by the price attribute which owned the highest degree of rational preference among consumers is also substantially higher, but lower than that of the diet cleanliness label attribute. The decoy effect caused by the breeding time attribute with the lowest degree of consumers’ rational preference is also the lowest. Therefore, the government should strengthen the publicity of the humane treatment of pigs before slaughter, and improve the certification and management system of pigs’ diet cleanliness. Besides, marketers are suggested to emphasis the product attributes that customer’s value the most in their advertising.

## Introduction

With the development of the economy and the improvement of social civilization, formulating and promoting corresponding animal welfare protection policies are inevitable. Animal welfare is a series of behaviors and external conditions that are provided for the well-being of animals. In many countries, especially in developed areas such as Europe and the United States, good animal welfare protection regulations have been incorporated by the government to reduce food safety risks. These regulations have become the norm for livestock and poultry feeding, transportation, slaughtering, and other aspects, and the public awareness of animal welfare protection is generally high (Wang and Gu, 2016). However, animal welfare is often a feature of a certain stage of the development of the agricultural economy. In China, although the pork traceability system was implemented 12 years ago, the requirements and safeguard measures for animal welfare are still in its infancy (Buller et al., 2018; Wang and Gu, 2020). To improve the quality and safety of pork and ensure the basic welfare of pigs, the “Farm Animal Welfare Requirements: Pigs” issued by Standards of China Association and the “National Live Pig Production Development Plan (2016–2020)” issued by the Ministry of Agriculture and Rural Affairs of the People’s Republic of China, clearly states that to ensure the quality and safety of pork, efforts should be made to improve animal welfare and resource utilization, so as to achieve the benign and sustainable development of the pork industry. However, in practice, it inevitably costs extra for producers to improve the animal welfare of pigs. Therefore, the standards and norms for the humane treatment of pigs have not been well implemented at the micro-production level (Ma, 2019). Only when the additional benefits of treating pigs humanely can compensate producers for the additional costs, will they be incentivized to effectively supply pork with high levels of animal welfare. The main benefit for producers to supply pork with high welfare standards are the price premiums paid by consumers (Vissers et al., 2019; Schröter and Mergenthaler, 2021). Accordingly, whether favorable animal welfare systems for pigs have economic advantages for consumers, or in other words, whether it can meet the utility of consumers and have exchange values, is a key factor in motivating producers to adopt meat production systems with higher animal welfare standards (Wang and Song, 2021). Therefore, understanding consumers’ preferences and their willingness to pay premiums for pork with different levels of animal welfare attributes will assist the government to scientifically formulate pork consumption policies in line with China’s actual market demand.

Studies in China and internationally mainly considered traditional economic theory of rational choice, that is, they assumed that consumers are completely rational, and will choose the most effective pork products according to their budget constraints (Lagerkvist and Hess, 2011; Clark et al., 2017). However, the context effect, based on modern decision theory, contradicts the traditional rational choice theory, according to which consumers are bounded rational during the actual purchase process, and various scenarios systematically impact their choices (Zhang and Du, 2016; Li et al., 2019). Although, based on the context effect of modern decision-making theory, some scholars studied the decoy effect of consumers’ purchasing behaviors of traceable pork in consumption scenarios with different inductive information, such as Liu and Chen (2019) and Wu et al. (2020). However, no scholars conducted comparative experimental research on rational and irrational consumption behaviors using the same sample. Scholars such as Janssen et al. (2016) and Wang and Gao (2020) reviewed the literature on empirical investigations of consumers’ purchasing behaviors and found that differences in survey objects had a significant impact on the survey results of consumers’ purchasing behaviors.

Therefore, conducting a survey and comparative study of consumers’ rational and irrational purchasing behaviors using the same sample can reduce the investigation bias caused by differences in survey objects, times, and regions, and more accurately reflect the similarities and differences between the rational and irrational purchasing behaviors of consumers. Therefore, this study considers pork, based on the rational choice theory in classical economics and the context effect in behavioral economics, and using the same survey sample, compares the internal connection between the rational preference order of consumers in the non-decoy environment and the irrational consumption behavior in the decoy environment. Furthermore, it explores whether irrational consumption violates the axiom of consumer’s rational preferences or causes a trade-off deviation of consumer’s choice behaviors. It is of great practical significance to scientifically identify consumers’ value trade-offs and preferences for pork with animal welfare attributes, thus effectively guiding consumers’ purchase behaviors and ensuring food safety in China.

## Literature review

This study reviews the related literature from three aspects: rational consumption and the related research methods, irrational consumption experiment and the decoy effect, and comprehensive research on rational and irrational consumption.

### Rational consumption and related research methods

The existing research on the consumption behaviors of livestock and poultry products with different attributes, is based on the traditional microeconomic theory, that is, the consumption behavior has completely rational characteristics and meets the axioms of completeness, reflexivity, and transitivity. The commonly used methods include Contingent Valuations and Conjoint Analysis. In Contingent Valuations, consumers’ overall value preferences for products are determined by creating a hypothetical market and direct inquiry, but it can easily lead to selection bias. It is a typical stated preference evaluation method. Studies mostly use this method, such as Rolfe (1999) and Bennett et al. (2002) to study the consumption preferences of Australian and British consumers for humanely treated laying hens but fail to measure the value of different animal welfare attributes. By contrast, Conjoint Analysis, through random combinations of different attribute levels of products, studies how consumers, under rational assumptions, choose the attribute combinations of a product to maximize utility based on budget constraints, and make value trade-offs for different attributes (McFadden, 1974). This method describes the product as a profile, each profile is composed of attributes and their different hierarchical combinations, which can describe the important features of the product. When consumers actually choose a product, they are not based on a certain attribute of the product, but comprehensively consider each attribute and their levels to make a purchase decision. Therefore, the method is highly dependent on the judgment ability of irrational consumers, and often takes a single consumption situation as the investigation background, while ignoring the impact of situational changes on consumers’ decision-making behavior. Existing conclusions about consumer preferences for pigs’ welfare in different countries are mostly drawn through this method. For example, Danish consumers value the living space attribute of pigs among different animal welfare attributes the most (Denver et al., 2017), German consumers value the free activity attribute of sows (Grunert et al., 2018), and the surgical anesthesia attribute of pigs (Latacz-Lohmann and Schreiner, 2019), Spanish consumers value pigs’ scatter-feed and injury-free attributes the most (García-Gudiño et al., 2021), Chinese consumers value the health aspect of animal welfare for pigs, such as timely treatment (Wu et al., 2020).

### Irrational consumption experiment and decoy effect

With the development of behavioral economics, people increasingly realize that consumers’ preferences and purchasing decisions are not completely rational, and that different scenarios will have different impacts on consumers’ decision-making behaviors, that is, the context effect. The decoy effect is a type of context effect. In this scenario, a decoy product C with attributes that are not superior to the target product B, and with at least one attribute that is superior to a competing product A is added into the selection set. Consumers obtain a decision reference point, and the probability of consumers selecting product B will increase, that is to say, a decoy effect occurs (Huber et al., 1982). Studies show that the intensity of the decoy effect is affected by factors such as the way that it is presented and the environment. For example, when the attributes of products such as televisions are represented by numbers or grades, it has a positive decoy effect on consumers’ purchasing behaviors (Frederick et al., 2014). Consumers in bar settings are more susceptible to decoy effects than consumers in library settings (Monk et al., 2016). Adding a fixed-fee decoy for deterministic payments and an extra-fee decoy for uncertain payments in the package service sector can increase the probability of the target scheme being selected (Zhang and Liu, 2017). Attributes reflecting food quality and safety have a strong decoy effect on consumers’ purchase of traceable pork (Liu and Chen, 2019).

However, some studies concluded that the decoys failed. For example, Attwood et al. (2020) used high-priced vegetarian products as a disadvantage decoy, and the results did not significantly increase the number of people choosing the target vegetarian product. The possible reason was that those consumers failed to fully perceive the attribute differences between the target and competitive products. The decoy products set by Ohlhausen and Langen (2020) based on the sales attributes even reduced the probability of consumers choosing the target product that is the resistance effect was produced. The possible reason was that the consumers had a conflict psychology. To the best of our knowledge, no empirical research on irrational consumption behaviors, when choosing livestock and poultry products with animal welfare attributes, has been conducted.

### Comprehensive research on rational and irrational consumption experiments

Scholars researched consumers’ purchasing decisions by comparing rational and irrational behaviors. They showed that, in addition to rationally calculating costs and benefits, consumers’ individual senses, emotions, habits, and other irrational factors also affected the final consumption results in terms of decision-making, judgments, and consumption behaviors. Therefore, consumers’ irrational behaviors, or bounded rational behaviors, always exist (Shi, 2016; Lee and Lee, 2018). The rational use of these irrational individual factors can induce consumers to make purchasing choices for healthy foods, such as eco-friendly foods (Kim et al., 2017). In addition, situational factors can also significantly affect the rational shopping behaviors of consumers and product scarcity and coincidence are the most typical situational factors (Akram et al., 2018).

Scholars including Denver et al. (2017), Grunert et al. (2018), Latacz-Lohmann and Schreiner (2019), and Wu et al. (2020), conducted extensive research on the consumer behaviors of animal products with animal welfare attributes. However, in the actual purchase behaviors, consumers’ choice decisions may also be affected by situational factors, such as the decoy effect. The experimental research on irrational consumption behaviors that introduces situational factors mainly focuses on common commodities, such as household appliances and service packages, and rarely pays attention to humane livestock and poultry products. In addition, few, if any studies consider these products by comparing the bounded rational consumers’ behaviors in traditional rational choice experiment and the irrational choice experiment with the introduction of decoys. Therefore, this study takes pork hindquarters as a specific research object and based on the reasonable consideration for animal welfare, sets three attributes which are breeding time, breeding mode, and diet cleanliness label to reflect animal welfare conditions and price attributes. It then compares consumers’ behaviors in rational and irrational consumption scenarios, explores the internal relationship between consumers’ preference order for different animal welfare information about pigs, and the decoy intensity of each piece of animal welfare information in irrational scenarios.

## Experimental design and research methods

### Settings of the experimental objects and their according attributes

Among meat products, Chinese consumers generally prefer to eat pork. In addition, China implemented a pork traceability system in 2010, which takes animal welfare traceability as one of the specific goals. Therefore, traceable pork was chosen as the experimental object in this article. To avoid the interference of different cuts of pork on consumer demand, a pre-survey was conducted. The results showed only small discrepancies in pork hindquarter prices in the different markets of the research area. Therefore, the experiment used pork hindquarter as a specific variety.

The overall utility of products to consumers mainly comes from the value trade-off of the various attributes of products (Lancaster, 1966). The study selects the product attributes and hierarchy of humanely raised pigs based on China’s national conditions. The current animal welfare problems of pigs in China are mainly concentrated on the excessively fast breeding, narrow and dirty enclosures, and swill feeding (Deng and Xiao, 2017). Therefore, in the experiments of rational and irrational consumption, three types of animal welfare attributes are considered, namely breeding time, breeding mode and diet cleanliness label with the corresponding levels, as shown in Table 1. According to preliminary research, in the large-scale pig farms in the experimental site, the growth time of pigs varies from 6 to 10 months. An overfly fast growth time can easily cause a burden on the heart and lungs of the pigs, which is not conducive to animal welfare (Mulder and Zomer, 2017). Therefore, the levels of breeding time attribute are set as *fast (6 months)* and *slow (10 months).* Pigs’ prolonged exposure to crowded, unventilated environments can lead to decreased immunity and spread bacteria (Wang et al., 2020), whereas free movement and expression of the animal’s natural instincts can reduce the risk of depression and immunosuppression. Therefore, the breeding modes for the pigs are set as *stall bred*^1^ and *free-range bred*^2^. To avoid bacterial infection caused by an unclean diet, the drinking water and feed must comply with China GB 5749 and NY/T 5027 specifications. Consumers prefer that food quality information is displayed on a label (Dopico et al., 2016), therefore, the diet cleanliness attribute levels of pork hindquarters are set as *with* and *without* these labels. This study assumes that the pork hindquarters are from China, and this was explained to consumers ahead of time. Referring to the average retail price of large supermarkets and e-commerce platforms in 2019, the price of domestic ordinary pork hindquarters was approximately 22 yuan/500 g. The humane pork hindquarters produced under the condition of suitable free-range breeding, and meeting the requirements of 10 months or more, has a relatively limited target market, and the price is approximately 40 yuan/500 g. Therefore, the price levels of pork hindquarters are set at *22, 31,* and *40 yuan/500 g*.

**Table 1:** The attributes of pork hindquarters and their corresponding levels.

Attributes	Corresponding levels
Breeding time	*Fast (6 months)*
	*Slow (10 months)*
Breeding mode	*Stall bred*
	*Free-range bred*
Diet cleanliness label	*Without*
	*With*
Price	*22 yuan/500 g*
	*31 yuan/500 g*
	*40 yuan/500 g*

### Experimental design of rational and irrational consumption behaviors

For the rational consumption experiment, the choice experiment method based on the rational person hypothesis was implemented to identify consumers’ preference order for pork hindquarters with different humane attributes without inductive information. The attributes and levels were randomly combined, using SSI Web 7.0 which is factorial design software. Fifteen different versions of the rational consumption experimental questionnaire were designed; each version included six selection cards for consumers to choose from. Additionally, to avoid bias in consumer decision-making, each selection card included the option: “I choose neither of them.” The D-efficiency of all the attributes was not less than 91.29%, indicating that the efficiency of the questionnaire design was at the highest level. A sample selection card is shown in Table 2.

**Table 2:** Example of a selection task for pork hindquarters.

Breeding time:	slow (10 months)	fast (6 months)	I choose neither of them
Breeding mode:	stall bred	free-range bred	
Diet cleanliness label:	with	without	
Price:	31 yuan / 500 g	22 yuan / 500 g	
**I would like to choose (only choose one)**	□	□	□

Considering that the choice experiment method based on the rational person assumption, which assumes that the consumer’s measurement of the utility of each attribute disregard the consumer’s contextual factors, which may cause the experimental results to deviate from market scenarios. Therefore, this study examines whether consumers’ preferences for pork with different humane attributes are irrational in the decoy scenarios, that is, whether there is a decoy effect. Following Liu and Chen’s (2019) method in the experiment of irrational consumption, we set pork hindquarters at low, medium, and high humane levels, which are represented by *a*, *b*, and *c* respectively, to form the core set $U\left\{a,b,c\right\}$ . Option *c* is the target product, and options *a* and *b* are the competing products. Four types of decoy options are set, which are represented by *d*, *e*, *f* and *g*, to form the expansion set $U_{1}\left\{a,b,c,d\right\},U_{2}\left\{a,b,c,e\right\},U_{3}\left\{a,b,c,f\right\},U_{4}\left\{a,b,c,g\right\}$ , as shown in Table 3. Among them, the decoy options *d*, *e*, *f*, and *g* had inferior attributes of breeding time, breeding mode, diet cleanliness label, and price, respectively, compared to the target option *c*. In the course of the specific survey, the number of times consumers choose option *c* in the core set and four extension sets is observed. Compared to the purchase share of the target product *c* in the core set, if the relative purchase share of it in the expansion set, increases in comparison to competitive products *a* and *b*, the attribute of the applicable decoy product has a positive decoy effect on consumers’ purchase behaviors.

**Table 3:** Consumption options for pork hindquarters with animal welfare.

Options in the core set	Fast (6 months), stall bred, without diet cleanliness label, 22 yuan/500 g (*a*)
	Fast (6 months), stall bred, with diet cleanliness label, 31 yuan/500 g (*b*)
	Slow (10 months), free-range bred, with diet cleanliness label, 40 yuan/500 g (*c*)
Decoy options	Fast (6 months), free-range bred, with diet cleanliness label, 40 yuan/500 g (*d*)
	Slow (10 months), stall bred, with diet cleanliness label, 40 yuan/500 g (*e*)
	Slow (10 months), free-range bred, without diet cleanliness label, 40 yuan/500 g (*f*)
	Slow (10 months), free-range bred, with diet cleanliness label, 44 yuan/500 g (*g*)

Finally, the questionnaire in this experiment is divided into three parts, including “individual characteristics of consumers,” “rational consumption experiment,” and “irrational consumption experiment.” By comparing consumers’ rational preference orders for humane attributes and the magnitude of the decoy effect in different attributes, we can assess whether there is an inherent relationship between decoy intensity and consumers’ preference orders in a non-decoy environment.

## Data sources and statistical description

The experiment was conducted in the city district of Wuxi, in the Jiangsu Province. Wuxi is located in the Yangtze River Delta, and its overall level of economic and social development is in a leading position in Jiangsu Province and even in China. According to the seventh national census data, Wuxi’s *per capita* Gross Demotic Product(GDP) in 2020 was 165,800 yuan, which ranked first in the country except for resource cities^3^. Residents’ food safety consumption consciousness and information demands are often directly proportional to their income and socioeconomic development levels (Li et al., 2019). In addition, Wuxi City was selected by the Ministry of Commerce in 2010 as one of the first pilot cities in which pork traceability was implemented. The traceability of the breeding section includes physiological, environmental, and sanitary animal welfare factors, therefore, Wuxi has a certain coverage of consumers with humane pork purchase experience, which can better meet the research conditions of consumption experiments. Not only that, the Wuxi municipal government has established a complete pork safety management system with a large local meat seller (Tianpeng Food Group), which has achieved good social benefits. The municipal government’s emphasis on food safety is at the forefront of the country. Therefore, conducting the research in Wuxi has a very suitable social basis. To ensure the representativeness of the experimental sample, trained master graduate students at a local university served as investigators, and the experimental participants were randomly recruited in all five administrative districts in the urban area of Wuxi. The third consumer who the investigator saw, was selected each time as a participant (Wu et al., 2012). For the sake of simplicity and convenience, 81 adult participants aged 18–65 were recruited in each district. In addition, illustrated posters were selectively placed to explain animal welfare to consumers. The experiment was completed in five batches from 1 to 20 June, 2020 and 405 valid questionnaires were obtained. To improve the enthusiasm of the participants, they were each given a small gift in gratitude if they completed the questionnaire.

Table 4 shows the sample statistical characteristics of the respondents. In this survey, women are the majority, accounting for 56.8% of the total sample, which is in accordance with Chinese customs that women mostly buy food for their family. The respondents were mostly younger than 40, accounting for 79.8% of the total sample. 77.6% of the respondents had a college or undergraduate degree, 60% had urban registered residence, and 82.7% earned from 3,000–12,000 yuan per month. In addition, the experiment used the Likert scale to explore Wuxi consumers’ confidence in the safety of meat and their cognition of animal welfare. The results showed that consumers were relatively satisfied with the current status of meat food safety; however, 15.5% of consumers were still a little worried or extremely worried about it. Consumers’ awareness of animal welfare was generally low with 83.2% of consumers selecting “low” or “very low” as the options for their knowledge of animal welfare.

**Table 4:** Statistical characteristics of respondents.

Statistical indicator	Category	Frequency (person)	Proportion (%)
Gender	Men	175	43.2
	Women	230	56.8
Age	18–30 Years old	213	52.6
	31–40 Years old	110	27.2
	41–50 Years old	69	17.0
	51–65 Years old	13	3.2
Educational background	Primary school and below	8	2.0
	Junior middle school	37	9.1
	Technical secondary school or high school	67	16.5
	Junior college	105	25.9
	Undergraduate course	169	41.7
	Graduate student or above	19	4.7
Registered residence	Urban	243	60.0
	Rural	162	40.0
Individual monthly income	≤3,000 yuan	45	11.1
	3,001–6,000 yuan	109	26.9
	6,001–8,000 yuan	147	36.3
	8,001–12,000 yuan	79	19.5
	>12,000 yuan	25	6.2
Attitude to the current state of meat	Extremely worried	9	2.2
	A little worried	54	13.3
	Neutral attitude	138	34.1
	Slightly satisfied	142	35.1
	Extremely satisfied	62	15.3
Knowledge about animal welfare	Very low	108	26.7
	Low	229	56.5
	Medium	58	14.3
	High	9	2.2
	Very high	1	0.2

## Model constructions in rational and irrational scenarios

In a no-decoy scenario composed of rational choice experiments, according to the consumer demand and random utility theories proposed by Lancaster (1966), the utility $U_{nit}$ obtained by the consumer n from the product i in the choice set C in scenario t consists of two parts, namely the deterministic utility $V_{nit}$ and the random utility $\varepsilon_{\mathrm{nit}}$ :


(1) $$U_{nit}=V_{nit}+\varepsilon_{nit}$$

Only when $U_{nit}\geq U_{njt}$ would consumer choose the pork hindquarters of type i . The probability of selection is expressed as follows:


(2) $$\begin{array}{l} \mathrm{Pr}\left(i\neq j\forall \in C\right)=\mathrm{Pr}(\beta_{1}X_{i1}+\beta_{2}X_{i2}+\cdots +\beta_{n}X_{in}\\ +\mu_{i}\geq \beta_{1}X_{j1}+\beta_{2}X_{j2}+\cdots +\beta_{n}X_{jn}+\mu_{j}) \end{array}$$

Among them, $X_{in}$ is the *n*th attribute of the *i*th pork hindquarter, and β is the utility score vector, representing individual preference. This study uses the Multinominal Logit (MNL) and Random Parameter Logit (RPL) models to estimate the consumers’ score utility results for each attribute level. The former needs to satisfy the strict independent identically distributed assumption of the random error term, while the latter relaxes this limitation and allows the parameters to change randomly among individuals, in other words, it allows consumers to have heterogeneous preferences and correlations between unobservable factors (Hu et al., 2004). The utility function is as follows:


(3) $$\begin{array}{l} {U_{nit}|}_{s}=chooseno+\beta_{s1}TIME_{nit}+\beta_{S2}MODE_{nit}\\ +\beta_{S3}LABEL_{nit}+\beta_{s4}PRICE_{nit} \end{array}$$

The attributes and levels of breeding time (TIME) , breeding mode (MODE) , diet cleanliness label (LABEL) , and the no-choice option (chooseno) used dummy code. When the corresponding product in the experiment is selected, the dummy code is one, and zero otherwise. The price attribute levels are continuous variables.

Consumers’ preference for each attribute is expressed by the relative importance of each attribute and calculated as follows:


(4) $$\begin{array}{l} \mathrm{Relative Importance}=\\ \frac{\left[\max\left(\beta_{i}\right)-\min\left(\beta_{i}\right)\right]\cdot \max\left(Z_{i}\right)}{\sum_{i=1}^{n}\left[\max\left(\beta_{i}\right)-\min\left(\beta_{i}\right)\right]\cdot \max\left(Z_{i}\right)}\cdot \mathrm{100}\% \end{array}$$

where, $Z_{i}$ represents the attribute level of the *i*^th^ attribute. According to the weighting principle of the relative importance of each attribute in the RPL model by Troiano et al. (2019), when the attribute adopts a virtual code, $Z_{i}$ =1, when the attribute is a continuous variable, such as the price attribute, $Z_{i}$ =10. The no-choice option is disregarded.

When in an irrational scenario, to determine the decoy effect value in the experiment, we referred to the calculation method of Mourali et al. (2007), set the core set as U{a,b,c} , where *c* is the competing product, and *a* and *b* are the target products. Set the expansion set after adding the decoy product *d* based on the breeding time attribute, as $U_{1}\left\{a,b,c,d\right\}$ . Set P(c;U) as the absolute purchase share of target product *c* in the core set U{a,b,c} . Set $P\left(a;U_{1}\right)$ , $P\left(b;U_{1}\right)$ , $P\left(c;U_{1}\right)$ , and $P\left(d;U_{1}\right)$ as the absolute purchase shares of the competing products *a* and *b*, target product *c*, and decoy product *d* in the expansion set $U_{1}\left\{a,b,c,d\right\}$ , respectively. Set $P_{d}\left(c;a:b\right)$ as the relative purchase share of target product *c* to the competing products *a* and *b* in the expansion set $U_{1}\left\{a,b,c,d\right\}$ after adding decoy product *d*.


(5) $$P_{d}\left(c;a:b\right)=\frac{P\left(c;U_{1}\right)}{P\left(a;U_{1}\right)+P\left(b;U_{1}\right)+P\left(c;U_{1}\right)}$$

At this point, the magnitude of the decoy effect is represented by ΔP :


(6) $$\Delta P=P_{d}\left(c;a:b\right)-P\left(c;U\right)$$

When ΔP>0 , a positive decoy effect occurs; when ΔP<0 , a negative decoy effect occurs.

## Results and discussion

### Consumers’ preference in a rational consumption experiment

**Figure 1:**
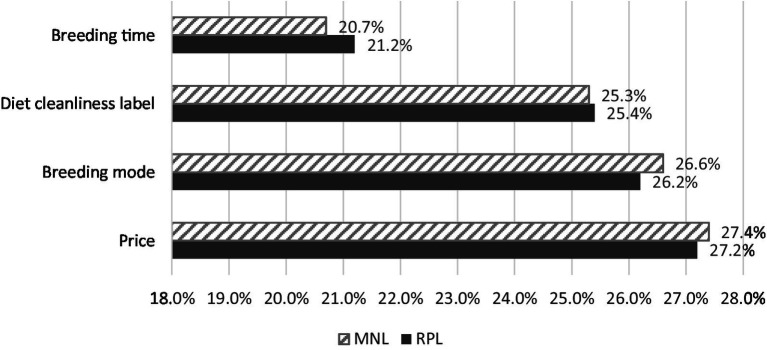
Estimation results of the relative importance of each attribute in the two logit models.

Using the NLogit5 measurement software, 2,430 samples (405 consumers × 6 selection cards) were processed, and the results of the RPL model were obtained as shown in Table 5. The score utility of the price attribute is negative and significant at the 1% level, indicating that the score utility of pork hindquarters to consumers has a significant inverse relationship with the price, which is consistent with the research of Wu et al. (2015) and Xu et al. (2019). That is, when the animal welfare attribute is the same, consumers perfer the cheaper one. The attributes score utility of breeding time, breeding mode, and diet cleanliness label are all positive and significant at the 1% level. This shows that compared to ordinary pork hindquarters, consumers prefer those produced under slow-growing, free-range, or clean-diet conditions. According to the utility score of each attribute in the MNL and RPL models and Equation (4), this study calculates the relative importance of the attributes of price, breeding mode, diet cleanliness label, and breeding time, as shown in Figure 1. According to the calculation results of the RPL model, the relative importance of the four categories of attributes is 27.2%, 26.2%, 25.4%, and 21.2%, respectively. This shows that consumers care most about price attributes, followed by animal welfare attributes. A possible reason is that meat that meets the requirements of animal welfare is expensive and many Chinese consumers are price sensitive (Tang, 2019; Xu et al., 2019), an increase in the beneficial attributes of products may become a burden; therefore many consumers will avoid these attributes for economic reasons. Furthermore, consumers are relatively unfamiliar with the listed animal welfare attributes, which causes them to concentrate more on the price.

**Table 5:** Parameter estimation results of the multinomial logit (MNL) and random parameter logit (RPL) models.

	MNL	RPL
Price	−0.167*** [−0.183, −0.151]	−0.185*** [−0.203, −0.167]
Breeding time	1.259*** [1.105，1.412]	1.443*** [1.224，1.663]
Breeding mode	1.623*** [1.471，1.776]	1.777*** [1.572，1.981]
Diet cleanliness label	1.544*** [1.390，1.696]	1.726*** [1.518，1.933]
No choice (Chooseno)	−2.243*** [−2.669, −1.818]	−2.504*** [−2.980, −2.028]
Standard deviation of random parameter distribution		
Breeding time	—	0.824*** [0.615，1.033]
Breeding mode	—	0.710*** [0.514，0.907]
Diet cleanliness label	—	0.653*** [0.447，0.860]
Pseudo residual square	0.291	0.329
Logarithmic likelihood value	−1850.951	−1789.099
Sample quantity	2,430	

^***^indicates significance at the 10% level.

In terms of consumers’ preference orders for the three welfare attributes, the relative importance of the breeding mode is the highest. Most likely the reason is that pork produced under free-range conditions is more tender than pork raised under space-constrained conditions, therefore, consumers prefer free-range pork. Apart from the attributes of breeding mode, consumers prefer the attribute of a diet cleanliness label, which may be affected by events such as clenbuterol-feeding and African swine fever in recent years, resulting in consumers’ preferring pork produced under safe diet conditions. Most consumers lack the scientific understanding of what a reasonable breeding time for pigs is, and may believe that raising pigs slowly reduces the tenderness of the meat or converts excess energy into fat (Zhang and Du, 2016), resulting in consumers preferring the slow-growing attribute of pigs the least.

### The change in consumer’s preference in the irrational scenarios

As shown in Table 6, in the core set U{a,b,c} , the absolute shares of consumers choosing pork hindquarters *a*, *b*, and *c* are 10.6, 31.9, and 57.5%, respectively. In the expansion set $U_{1}\left\{a,b,c,d\right\}$ after adding the breeding time attribute decoy *d*, the absolute share of consumers choosing pork hindquarter *c* is 64.0%. In the expansion set $U_{2}\left\{a,b,c,e\right\}$ after adding the breeding mode attribute decoy *e*, the absolute share of consumers choosing pork hindquarter *c* is 64.9%. In the expansion sets $U_{3}\left\{a,b,c,f\right\}$ and $U_{4}\left\{a,b,c,g\right\}$ after adding the diet cleanliness label attribute decoy *f* and the price attribute decoy *g*, the absolute share of consumers choosing pork hindquarter *c* is 68.4 and 67.4%, respectively. Therefore, it can be seen that different decoy products have different utility strengths in influencing consumers’ purchasing decisions.

**Table 6:** The absolute purchase share of each product in irrational scenarios.

Pork Hindquarter	Core set *U*	Expansion set *U*_1_	Expansion set *U*_2_	Expansion set *U*_3_	Expansion set *U*_4_
*a*	43(10.6%)	39(9.6%)	39(9.6%)	33(8.1%)	32(7.9%)
*b*	129(31.9%)	86(21.2%)	85(21.0%)	79(19.5%)	81(20.0%)
*c*	233(57.5%)	259(64.0%)	263(64.9%)	277(68.4%)	273(67.4%)
*d*	—	21(5.2%)	—	—	—
*e*	—	—	18(4.5%)	—	—
*f*	—	—	—	16(4.0%)	—
*g*	—	—	—	—	19(4.7%)

Please see Table 3 for the explanation of the letters *a*–*g*.

As shown in Table 7, according to Equations (5) and (6), the relative share of consumers purchasing pork hindquarter *c* increased from 57.5% in the core set *U* to 67.5%^4^ in the expansion set *U_1_*, $P\left(c;U\right)<P_{d}\left(c;a:b\right)$ , and ΔP of the decoy effect is 10.0%. Therefore, the breeding time attribute has a positive decoy effect on consumers’ purchasing behaviors. The same observation applies to the core set *U* and expansion sets *U*_3_ and *U*_4_, where the relative share of pork hindquarters purchased by consumers increased to 71.3 and 70.7%, respectively. The magnitude ΔP of the decoy effect was 13.7 and 13.2%, respectively. Therefore, the attributes of a diet cleanliness label and price also have positive decoy effects on consumers’ purchasing behaviors in choosing pork.

**Table 7:** The purchase share of pork hindquarter *c* relative to *a* and *b.*

	Core set *U*	Expansion set *U*_1_	Expansion set *U*_2_	Expansion set *U*3	Expansion set *U*_4_
P(*c*;*U*)	57.5%	—	—	—	—
P*_i_*(*c*;*a*,*b*) (*i* = *d*,*e*,*f*,*g*)	—	67.5%	68.0%	71.3%	70.7%
ΔP	—	10.0%^*^ (31.17)*p*	10.5%^*^ (29.06)*p*	13.7%^*^ (33.13)*p*	13.2%^*^ (34.75)*p*

Please see Table 3 for an explanation of the explanation of the letters *a*– *g*; In parentheses p is $\chi^{2}\left(3\right)$ .

* indicates significance at the 1% level.

Thus, in the pig hindquarter consumption experiment, the four kinds of decoys are effective, and the intensity order is the diet cleanliness label, price, breeding mode, and breeding time. In this regard, a reasonable explanation is that the outbreak of events of clenbuterol-feeding and African swine fever in recent years resulted in Chinese consumers being concerned about meat safety. Guaranteeing that pigs have clean diets prevents pork safety risks; therefore, the decoy effect of the attribute of diet cleanliness label is the strongest. The intensity of the decoy effect of the price attribute is only in second place. In contrast, in the rational purchase experiment of pork hindquarters, consumers attached the most importance to the price attribute. Therefore, in the process of promoting humane pork, the price attribute also plays a role that cannot be underestimated (Lai et al., 2017; Wu et al., 2020). Although the attributes of breeding mode and time can reflect the level of pork safety and animal welfare, these attributes are not intuitively attributed to food safety in the same way as a clean diet label. Therefore, these two attributes’ abilities to induce consumers to choose the targeted pork hindquarters are relatively weaker.

### Comparison of the results of consumers’ rational and irrational consumption experiments

**Figure 2:**
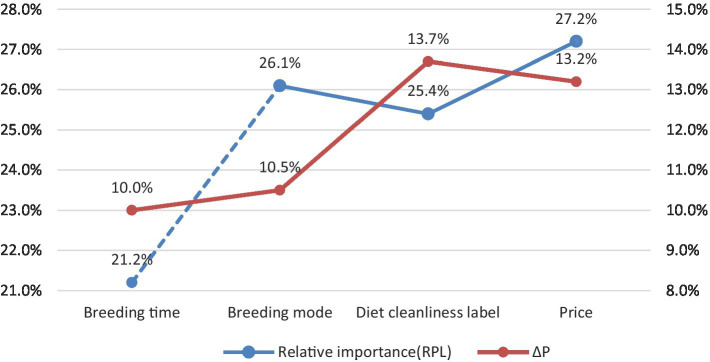
The relative importance and the decoy effect of each attribute.

In this study, the relative importance of animal welfare attributes in rational consumption experiments is compared to the intensity of decoy effects in irrational consumption experiments to explore the internal relationship between the two. The overall trend of the two sets of data is consistent, indicating that there are certain similarities and differences between the relative importance of animal welfare attributes and the changes in the intensity of the decoy effects. Although the purchase behaviors in the irrational consumption scenarios violate the basic preference axiom of the rational person hypothesis, the trade-off bias caused by this does not completely negate the experimental results of the traditional rational consumption. In the no-decoy environment, the relative importance of the breeding time attribute is the lowest, and the relative importance of the price attribute is the highest for consumers. Then, in the decoy scenario, the effect level of the decoy product set based on the breeding time attribute is the lowest, and that of the decoy product set based on the price attribute is relatively high but not the highest. In the no-decoy environment, consumers’ preference for the attribute of a diet cleanliness label is only slightly higher than that of breeding time, whereas it is the highest in the decoy environment. It can be seen that consumers are most vulnerable to the decoy effect of a diet cleanliness label which can facilitate avoiding food safety risks. This is similar to the research results of Liu and Chen (2019), that is, in the irrational consumption behaviors of traceable pork, consumers are more susceptible to decoys with the utility of avoiding food safety risks (Figure 2).

## Conclusion and suggestions

The article considers a food product, pork hindquarters, and sets attributes of breeding time, breeding mode, diet cleanliness label and price, to assess consumers preference orders for each attribute in a no-decoy scenario, and compare with the decoy effect intensity in a decoy scenario, thus exploring the inner connection between rational and bounded rational consumption behaviors of pork with animal welfare attributes.

The first conclusions reveals that the surveyed consumers’ awareness of animal welfare is generally low, and approximately 80% of consumers do not understand the concept and connotation of animal welfare, which is not conducive to promoting and popularizing pork with high animal welfare attributes.

Second, Under the rational consumption circumstances without decoy scenarios, the relative importance of the four types of animal welfare attributes, to consumers, is ranked as follows: price (27.2%), breeding mode (26.2%), diet cleanliness label (25.4%), and breeding time (21.2%).

Third, under the irrational consumption circumstances with decoy scenarios, the order of the decoy effect of the four types of animal welfare attributes on consumers is the diet cleanliness label (13.7%), price (13.2%), breeding mode (10.5%), and breeding time (10.0%).

Fourth, comparing the results of rational and irrational consumption experiments, consumers are most easily influenced by the decoy with a diet cleanliness label because of their concerns for food safety. The decoy effect caused by the price attribute with the highest consumers’ preference is also higher, but lower than that of the diet cleanliness label. The breeding time attribute with the least rational consumer preference has the lowest decoy effect. Therefore, the deviation of consumers’ purchasing behaviors caused by irrational consumption scenarios does not completely negate the results of traditional rational consumption experiments.

Accordingly, countermeasures and suggestions are proposed.

1. Strengthen consumer education. The government and production enterprises can strengthen the publicity of the welfare attribute through radio, WeChat public account, community publicity, or other channels so that consumers can better understand the role of the length of breeding time, different breeding modes, and diet cleanliness in improving pork quality and ensuring pork safety, thus guiding consumers to purchase traceable pork with animal welfare attributes.
2. Establish a certification system for pigs’ safe and healthy diets. Consumers’ preference for the attribute of clean diets is second only to the attribute of the breeding mode. Therefore, government departments can formulate a corresponding certification system for safe and healthy diets, and guide farmers and producers to have their pigs’ diets certified, which will improve the welfare of pigs and meet consumers’ diverse needs for pork products.
3. Reasonable marketing. Considering that the relative importance of animal welfare attributes is closely related to the intensity of the decoy effect, the production and marketing enterprises should pay more attention to the attributes valued by consumers when promoting, and reasonably formulate marketing promotion strategies for humane pork. For example, under the premise of policy permitting, putting into some pork with nearly the same quality but have inferior diet cleanliness attribute or relatively unfriendly price attribute for consumers to compare, which can effectively guide them to purchase pork with high animal welfare, thereby stimulating the market potential of traceable pork with humane attributes.

This study also has certain limitations. First, the method of comparative choice experiment adopted in this study is a hypothetical experiment without real monetary transaction behaviors, and experimental participants’ stated preferences for products may deviate from their actual consumption choices, especially in rational consumption experiments, they may have a psychological tendency to exaggerate their willingness to pay (Gracia et al., 2011; Penn and Hu, 2018). In addition, in the expansion set in irrational consumption experiment, there are as many as two competing products except to the target product and the decoy product, which may interfere the identification of consumers of dominant products and limit the strength of the decoy effect. Finally, the experiment only based on one developed city in China, therefore the universality of the experimental conclusions needs to be further verified in other cities.

## Data availability statement

The raw data supporting the conclusions of this article will be made available by the authors, without undue reservation.

## Ethics statement

The studies involving human participants were reviewed and approved by The ethics committee of Jiangnan University. The patients/participants provided their written informed consent to participate in this study.

## Author contributions

LX proposed the research direction, designed the structure of the article, and wrote the manuscript. MY designed the questionnaire and analyzed the data. XC revised the manuscript. All authors contributed to the article and approved the submitted version.

## Funding

This work was supported by the Humanities and Social Sciences Planning Foundation Project of the Ministry of Education (grant no. 20YJA790076) and the National Natural Science Foundation of China (grant no. 71803067).

## Conflict of interest

The authors declare that the research was conducted in the absence of any commercial or financial relationships that could be construed as a potential conflict of interest.

## Publisher’s note

All claims expressed in this article are solely those of the authors and do not necessarily represent those of their affiliated organizations, or those of the publisher, the editors and the reviewers. Any product that may be evaluated in this article, or claim that may be made by its manufacturer, is not guaranteed or endorsed by the publisher.

## Footnotes

^1^Pigs are raised separately; the size of the stall is approximately 2.2 m (length) × 70 cm (width), which is only slightly larger than the size of a pig. The pigs cannot move freely.

^2^This was adopted to raise ecological white pigs; approximately 5–8 pigs are raised per 100 m^2^. The pigs can move freely.

^3^"*The power of Wuxi, lifting trillions of GDP*," [Accessed 9/14/2021], Wuxi Municipal People ‘s Government official website, Available at: http://www.wuxi.gov.cn/doc/2021/09/14/3423606.shtml.

^4^According to Equation (5), $P_{d}\left(c;a:b\right)=\frac{1}{9.6\%+21.2\%+64.0\%}=67.5\%.$
